# Differences between Total Intravenous Anesthesia and Inhalation Anesthesia in Free Flap Surgery of Head and Neck Cancer

**DOI:** 10.1371/journal.pone.0147713

**Published:** 2016-02-05

**Authors:** Yi-Ting Chang, Chih-Chen Wu, Tsung-Yung Tang, Chun-Te Lu, Chih-Sheng Lai, Ching-Hui Shen

**Affiliations:** 1 Department of Anesthesiology, Taichung Veterans General Hospital, Taichung, Taiwan; 2 Division of Plastic and Reconstructive Surgery, Department of Surgery, Taichung Veterans General Hospital, Taiwan, ROC; 3 School of Medicine, National Yang-Ming University, Taipei, Taiwan; Scientific Inst. S. Raffaele Hosp., ITALY

## Abstract

**Background:**

Many studies have evaluated risk factors associated with complications after free flap surgery, but these studies did not evaluate the impact of anesthesia management. The goal of the current study was to evaluate the differences between patients who received inhalation and total intravenous anesthesia (TIVA) in free flap surgery.

**Methods:**

One hundred and fifty-six patients who underwent free flap surgery for head and neck cancer were retrospectively divided into the TIVA (96 patients) and the inhalation group (87 patients). Perioperative hemodynamic data and postoperative medical complications were determined by documented medical records.

**Results:**

Ninety-six patients in the TIVA group were compared with 87 patients who received inhalation anesthesia. There were no differences in gender, age, classification of physical status based on American Society for Anesthesiologists (ASA) score, and cormobidities between the two groups. Patients in the TIVA group required less perioperative crystalloid (4172.46 ± 1534.95 vs. 5183.91 ± 1416.40 ml, *p* < 0.0001) and colloid (572.46 ± 335.14 vs. 994.25 ± 434.65 ml, *p* < 0.0001) to maintain hemodynamic stability. Although the mean anesthesia duration was shorter in the TIVA group (11.02 ± 2.84 vs. 11.70± 1.96 hours, *p* = 0.017), the blood loss was similar between groups (*p* = 0.71). There was no difference in surgical complication rate, but patients in the TIVA group developed fewer pulmonary complications (18 vs. 47, *p* = 0.0008). After multivariate regression, patients in the TIVA group had a significantly reduced risk of pulmonary complication compared with the inhalation group (Odds ratio 0.41, 95% CI 0.18–0.92).

**Conclusions:**

Total intravenous anesthesia was associated with significantly fewer pulmonary complications in patients who received free flap reconstruction.

## Introduction

Microvascular free tissue flap reconstruction has been performed widely by otolaryngologists and plastic surgeons since the 1970s and has become the gold standard for surgical treatment of head and neck cancer. The overall success rate is 95% ~99.2%[1]. However, despite the high success rate, postoperative medical and surgical complications of major head and neck tumor ablation with free flap reconstruction do occur. Previous research showed the overall medical complication rate was between 27.6%[2] and 32.5%[3]. Clark et al. reported several medical complications including myocardial infarction, congestive heart failure, stroke, pneumonia, and multiorgan failure[2]. Among the medical complications, postoperative pulmonary complication was most common and was correlated with prolonged hospitalization and time spent in the intensive care unit[4]. Moreover, a variety of factors related to surgical complication were also found, including tracheostomy, preoperative hemoglobin (<11 g/dL) and the preoperative radiotherapy[2].

Previous research has suggested that the maintenance of hemodynamic stability and adequate perfusion for fresh anastomosis during free flap reconstruction is crucial in the anesthestic management of free flap surgery[5]. The primary focus of these studies was high cardiac output, systolic blood pressure > 100 mmHg, low systemic vascular resistance and high urine output. However, the anesthetic management varies and, even though it is based on physiological principles, more documentation is needed[6, 7].Inhalation anesthetic as well as total intravenous anesthesia(TIVA) have been used worldwide for decades in the maintenance of anesthesia.However, there is no consensus on perioperative management, which may affect the final outcome. The present study aimed to evaluate the difference between TIVA and inhalation groups and to determine the impact of anesthesia management on postoperative complications in free flap surgery of head and neck.

## Methods

### Study setting and database

**Fig 1:**
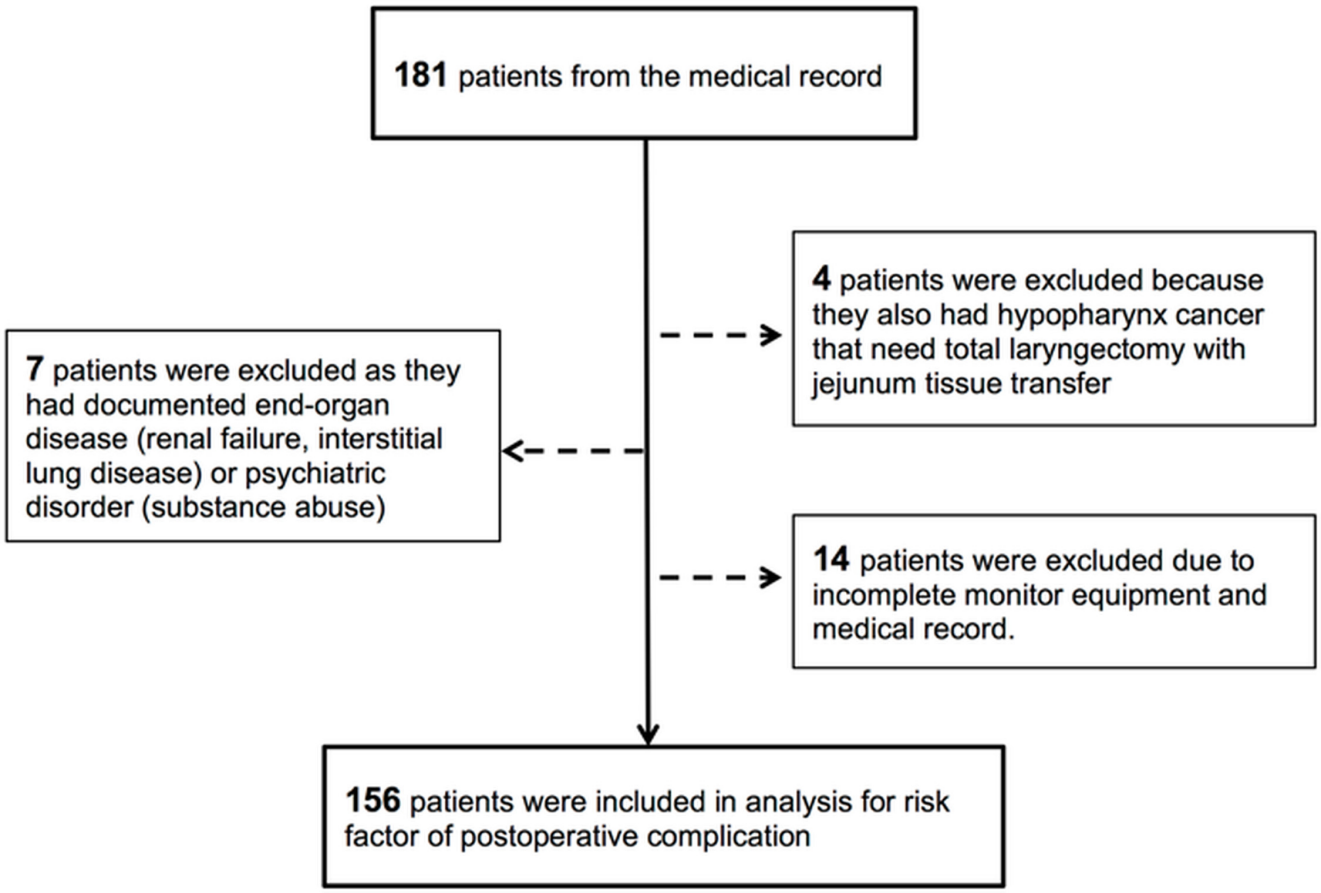
Flow chart of the retrospective study. One hundred and eighty patients were enrolled from medical records using key words “free flap reconstruction”. Four patients were excluded because they also had hypopharynx cancer that needed not only free flap reconstruction but also jejunum tissue transfer. Seven patients were excluded because they either had end-organ disease or a psychiatric disorder that may have complicated their recovery course. Finally, fourteen patients were excluded due to incomplete medical records. A total 156 of patients were included in the final analysis.

The study protocol was approved by the Institutional Review Board (IRB) of Taichung Veterans General Hospital. Because the study was designed as a retrospective research of medical records and was certified by the IRB as low risk, informed consents was not required by the IRB. Medical records of patients who received free flap reconstruction for major head and neck cancer surgery, which was performed by two senior plastic surgeons at a single medical center between January 2012 and October 2013 were collected by a single researcher. All patients who received tumor excision and free flap reconstructions for head and neck cancer during the period were included. The clinicopathologic data, including demographic, comorbidities, as well as surgical and anesthetic variables were obtained from medical charts. All patients received operation under the combined departments of Otolaryngology and Plastic Surgery. A total of 181 cases were included initially. Exclusion criteria were as follows: patients with hypopharyngeal cancer who needed total laryngectomy with jejunum tissure transfer, those with documented end-organ disease, psychiatric history, and patients with incomplete records of perioperative monitoring equipment use (arterial catheter for real-time arterial pressure measurement and central venous catheter for intravenous fluid application and intravascular volume monitor). After 25 patients were excluded using these criteria, 156 patients remained for further analysis (Fig 1). Prior to analysis, patients’ information were anonymized and de-identified by the same researcher who collected data from medical records.

### Perioperative management

Anesthesia was introduced by intravenous administration of 2ug/kg Fentanyl, 1mg/kg Lidocaine, 1-2mg/kg Propofol and 0.8–1.2mg/kg Rocuronium. If the patient was identified as having difficulty with intubation, we performed flexible fiberoptic intubation under intravenous sedation with 0.1mg/kg Midazolam. After the endotracheal tube was inserted, anesthesia was maintained with inhalation anesthetic (50% O_2_/Air with Sevoflurane 2% or Desflurane 8%) or TIVA using Target-controlled infusion(TCI) system (Orchestra® Base Primea; Fresenius Kabi, Germany) based on the pharmacokinetics model proposed by Schnider et al. with a target effect-site concentration of 3.0~5.0mcg/ml in accordance with the attending anesthesiologists’ preferences. We set up routine hemodynamic monitoring including electrocardiography, oximetry, NIBP, as well as arterial and central venous catheterization.

The goal of perioperative management was to achieve hemodynamic stability in order to maintain adequate perfusion pressure on the freshly created graft. When a hypotension episode occurred during the surgery, the anesthetic staff were given priority to begin intravenous fluid resuscitation and blood product transfusion rather than using a vasopressor to avoid compromising the anastomosis of the vessel. After the operation, patients were transferred to the post-anesthesia care unit (PACU) with mechanical ventilation. If patients were awake and anxious, they were sedated using Midazolam with a dose 20-100mcg/kg/hr, following the protocol of sedation of ventilated patients in both groups in order to prevent possible agitation which would possibly compromise the microvascular anastomosis. The goal of sedation was to maintain patients' cooperative, orientated and tranquil status, with a Ramsay scale of 2. All patients received at least one chest radiography as soon as they arrived at the PACU for postoperative follow-up. At 6:00 AM on postoperative day 1, sedation was discontinued and pressure support mode was applied for at least one hour before patients were separated from the ventilator.

### Patient variables

We reviewed all the data of patients undergoing head and neck surgery followed by free flap reconstruction. Demographic data, comorbidities, anesthesia variables, perioperative hemodynamic parameters and surgical variables were recorded by a single researcher. Patients and perioperative hemodynamic characteristics of interest were those considered to be potential predictors of postoperative complications based on the guidelines suggested in the literature review[2, 6, 8].

### Outcome variables

The outcome variables of the study were the incidences of postoperative medical and surgical complications. Postoperative medical complications included cardiovascular events (documented myocardial ischemia such as abnormal ECG change, elevated cardiac enzyme, or coronary vessel stenosis by cardiac catheterization), cerebrovascular events (documented stroke from computed tomography), and pulmonary events (documented pulmonary edema, pneumonia or atelectasis based on the reports of routine postoperative chest radiography by radiologists.) Postoperative surgical complications were based on documented data from the electric medical record system which included thrombosis, bleeding, and graft failure that needed repeat operation after initial free flap surgery.

### Statistics

All statistical analysis was performed using SPSS statistical software (IBM® SPSS Statistics 22.0). Categorical variables were compared using Pearson’s chi-square test, and the Fisher’s exact test was used when the frequency was expected to be small. For continuous variables, we performed Kolmogorov-Smirnov test to confirm normality. Independent t-test was used for normally distributed continuous variables and Mann-Whitney test was used for non-parametric continuous variables. Variables with a *p* value less than 0.05 were considered statistically significant. Parameters with *p* value less than 0.1 in the univariate logistic regression were entered into the multivariate logistic regression model using enter selection.

## Results

### Patient demographic and comorbidites

Among 156 patients, male gender was predominant (94.2%). The mean age of patients was 52.42±9.78 years. The physical status of most patients was categorized as Americam Society fo Anesthesiologists physical status (ASA) class II (n = 129) (82.7%). Forty-seven patients (31.91%) were current smokers, 52 patients were diagnosed with hypertension (32.62%), 24 patients presented with diabetes mellitus (16.31%). Sixty-nine patients(44.2%) received TIVA as the maintenance anesthesia and 87 patients(55.8%) received inhalation anesthetic.The mean anesthesia duration was 11.40 (± 2.40) hours. Hemodynamic data including central venous pressure (CVP), urine output, the quantity of intravenous fluid administrated, intraoperative blood loss and overall fluid balance are shown in Table 1.

**Table 1:** Clinical and demographic characteristics(Total patients = 156).

**Patient variables**		**Anesthesia variables**	
Male, no. (%)	147 (94.2)	TIVA, no (%)	69 (44.2)
Age, yr	52.42 ± 9.78^a^	Inhalation, no(%)	87 (55.8)
BMI, kg/m^2^	23.84 ± 4.04 ^a^	Anesthesia duration, hr	11.40 ± 2.40 ^a^
ASA I, no(%)	11(7.1)	**Outcome variables**	
ASA II, no(%)	129(82.7)	Inhospital mortality	2(1.3)
ASA III, no(%)	16(10.3)	Overall complication, no. (%)	105(33.65)
Smoker, no	47 (30.1)	Surgical complication, no	40
Preop C/T, no (%)	53 (34)	Medical complication,no	65
Preop R/T, no (%)	52 (33.3)	Cardiovascular,no.(%)	0
Hypertension	52 (33.3)	Cerebralvascular, no(%)	0
Diabetes mellitus	24 (15.4)	Pulmonary, no. (%)	65

BMI, Body mass index; ASA, Smoker, current smoker at the time of admission; Preop C/T, Preoperative chemotherapy; Preop R/T, Preoperative radiotherapy; TIVA, Total Intravenous anesthesia

^a^ Continuous variables were present with mean ± standard deviation.

### Mortality

Two patients in the inhalation group died during hospital stay. Both of them were categorized as ASA class II.One was a 38-year-old male who died 14 days after the surgery because of bronchospasm-related hypoxic encephalopathy. The other one was a 58-year-old male who died from severe hospital-acquired pneumonia. The total mortality was 1.3%.

### Overall complication

Sixty-five medical complications and 40 surgical complications developed after the surgery. None of the 65 medical complications were cardiovascular or cerebrolvascular. Therefore, further analysis of medical complications focused on postoperative pulmonary complications.

### Differences between groups (TIVA versus inhalation anesthesia)

There were no differences between the two groups in cormobidities including gender, age, BMI, ASA class, smoking history, preoperative chemotherapy or radiotherapy, hypertension and diabetic mellitus(Table 2). In comparison with patients who received inhalation anesthetic, patients who received TIVA had shorter duration of anesthesia (*p* = 0.017), significantly lower mean CVP (*p* = 0.0039), lower urine output (*p* = 0.0001), lower quantity of intravenous fluid administered with crystalloid(*p* < 0.0001) and colloid (*p* < 0.0001), less overall fluid balance(*p* < 0.0001) (Table 2).

**Table 2:** Comparison between the TIVA and inhalation groups.

	TIVA group (n = 69)	Inhalation group (n = 87)	*p* value
**Patient variables**			
Male, no.	64	83	0.510
Age, yr	52.80±10.93	52.11±8.82	0.667
BMI, kg/m^2^	24.07 ±4.00	23.66 ± 4.09	0.325
ASA class III	9	7	0.426
Smoker, no	22	25	0.803
Preop C/T	19	34	0.180
Preop R/T	22	30	0.864
Hypertension	24	28	0.864
Diabetes mellitus	10	14	0.959
**Anesthesia variables**			
Mean CVP, mmHg	7.04 ± 3.13	8.53 ± 3.23	0.0039
Total U/O, ml	1509.86 ± 1063.95	2074.48 ± 1115.10	0.0001
Total crystalloid, ml	4172.46 ± 1534.95	5183.91 ± 1416.40	< 0.0001
Total colloid, ml	572.46 ± 335.14	994.25 ± 434.65	< 0.0001
Blood loss, ml	918.77 ± 656.84	859.71 ± 486.47	0.707
I/O, ml	2316.30 ± 1056.73	3243.97 ± 1282.99	< 0.0001
Anesthesia duration, hr	11.02 ± 2.84	11.70± 1.96	0.017

BMI, Body mass index; ASA, classification of American society for anesthesiologist physical status; Smoker, current smoker at the time of admission; Preop C/T, Preoperative chemotherapy; Preop R/T, Preoperative radiotherapy; TIVA, Total Intravenous anesthesia; CVP, Central venous pressure; U/O, urine output;

I/O, total fluid balance; Significant at *p* < 0.05 level

### Postoperative surgical complication

Nineteen patients from the TIVA group and 21 patients from the inhalation group developed surgical complications. However, there was no significant difference between the two groups.(Table 3)

**Table 3:** Postoperative outcome between TIVA and inhalation groups.

	TIVA group (n = 69)	Inhalation group (n = 87)	*p* value
**Outcome variables**			
Pulmonary complication, no	18	47	**0.0008**^**a**^
Surgical complication, no	19	21	0.766
Ventilator duration, hr	30.27 ± 51.80	34.49 ± 46.16	0.395
ICU stay, days	3.87 ± 4.03	4.14 ± 2.08	0.082
Hospital stay, days	20.54 ± 9.61	23.59 ± 12.12	0.113
Inhospital mortality	0	2	0.503

^**a**^ *p* < 0.05 as significant; NS, nonsignificant

### Postoperative pulmonary complication

Sixty-five patients developed postoperative pulmonary complication with an overall postoperative pulmonary complication rate of 41.7%. Patients in the TIVA group had significantly fewer postoperative pulmonary complications (18 vs. 47, *p* = 0.0008)(Table 3.).Because of the differences between the inhalation and the TIVA group, including anesthesia duration, mean CVP, total urine output and the volume of crystalloid and colloid administration,we had adjusted these covariates by multiple regression analysis.There were significantly fewer pulmonary complications in the TIVA group compared with the inhalation group. (Odds ratio 0.41, 95% CI: 0.18–0.92, *p* = 0.031) in the multivariate logistic regression analysis(Table 4).

**Table 4:** Regression of pulmonary complication.

Variable	Univariate	Multivariate	Odds ratio	95% CI
	*p* value	*p* value		
Anesthetics (TIVA)	0.0004^a^	0.031^b^	0.41	0.18–0.92
Mean CVP	0.357	-	-	
Total U/O, ml	0.648	-	-	
Total crystalloid, ml	0.163 ^a^	-	-	
Total colloid	0.005 ^a^	0.455		
Blood loss, ml	0.347	-	-	
I/O, ml	0.006 ^a^	0.274		
Anesthesia duration, hr	0.019 ^a^	0.211		

^a^Variables enrolled into multivariate regression analysis if a univariate *p* < 0.1

^b^ *p* < 0.05 as significant; NS, nonsignificant

## Discussion

The demographic data of our study were similar to those of previous studies, which suggested that head and neck patients are predominatly male, often have a history of smoking, and are more likely to have comorbidites such as hypertension and diabetes mellitus[2, 3]. Compared to the patients in a study by Clark and colleagues, most of our patients were ASA class I& II (89.7% vs. 49.2%[2], respectively) and younger (mean age 52.64 years vs. 60.1 years[2]). These two factors may account for the absence of medical complications such as myocardial ischemia, congestive heart failure and stroke in our patients. The total mortality in our study was 1.3%, which was comparable with that of other studies (le Nobel et al.[3], 0.3% and the Clark et al.[2], 1.6%). In our study, 105 complications (65 medical and 40 surgical) developed after surgery. The overall complication rate was similar to that of a previous study[3].(33.65% vs. 32.6%).

This study analyzed 156 patients with head and neck cancer who underwent free flap surgery. Comparing the 96 patients who received TIVA to the 87 patients who received inhalation anesthesia, there were no differences in gender, age, classification of American Society for Anesthesiologists physical status (ASA),BMI,current smoking history, preoperative chemotherapy, preoperative radiotherapy or cormobidity such as hypertension and diabetes mellitus between two groups. Patients in the TIVA group needed significantly less intravenous crystalloid (4172.46 ± 1534.95 vs. 5183.91 ± 1416.40 ml, *p* < 0.001) or colloid (572.46 ± 335.14 vs. 994.25 ± 434.65 ml,*p* < 0.0001) administration to maintain hemodynamic stability which may have resulted in significantly lower central venous pressure (7.04± 3.13 vs. 8.53 ± 3.23 mmHg, *p* = 0.0039), less urine output (1509.86 ± 1063.95 vs. 2074.48 ± 1115.10 ml, *p* = 0.0001), and less total fluid balance(2316.30 ± 1056.73 vs. 3243.97 ± 1282.99 ml, *p* < 0.0001).

The goal of anesthesia for free flap transfer is to keep adequate perfusion of the flap, including maintenance of a high cardiac output, systolic blood pressure over 100 mmHg, relatively low systemic vascular resistance, together with a mild hemodilution to achieve a hematocrit value of 30–35%[6]. The reason for less fluid requirement to maintain hemodynamic stability in the TIVA group may be explained by a study published by Sudheer[9], which showed a relative cardiovascular stability in the propofol group because of the significantly increased SVRI compared with the inhalation group in position change. Deryck et al.[10] found propofol did not affect the left ventricle afterload or ventricular performance, while sevoflurane decreased arterial vessel tone and reduced left ventricular-arterial coupling. We postulate that the milder modulation on vascular resisitance lead to significantly lower perioperative fluid requirement in the patients who received TIVA as the maintenance anesthesia compared with patients who received inhalation anesthetic.

The most important finding in this study was that patients in the TIVA group had significantly associated with fewer postoperative pulmonary complications(18 vs.47, *p* < 0.0008) (Table 3). In deed, it is reasonable to speculate that owing to the significantly lower perioperative fluid requirement in the TIVA group, less pulmonary complication developed postoperatively. Several studies suggested repeated fluid resuscitation could lead to increased postoperative medical complications[1, 2]. The finding was in line with that of a study by Zhong et al[11].that showed crystalloid was an independent risk factor for postoperative complications and suggested the crystalloid volume replacement rate should be between 3.5 and 6 ml/kg/hr. The difference of overall fluid balance may also lead to fewer pulmonary complications. In this study, overall fluid balance in the TIVA group (2316.30 ± 1056.73 ml) was significantly lower than that in the inhalation group, which was similar to the results of a recently published national survey in the United Kingdom[7].

Despite the obvious differences in fluid requirement, the anesthesia duration was shorter in the TIVA group(11.02 ± 2.84 vs. 11.70± 1.96 hours, *p* = 0.017). Patients with a more serious medical condition such as poor nutrition status and impaired cardiopulmonary function may not to be able to tolerate prolonged surgery and would be more likely to develop complications. The longer duration of anesthesia may be correlated with a more challenging and complicated operation along with greater intraoperative fluid shifts, which would tend to make patients vulnerable to pulmonary complications following aggressive fluid administration. However, the blood loss was similar between groups (*p* = 0.71) in the current study. In order to determine wheather the shorter anesthesia duration or TIVA was correlated with lower pulmonary complication, we adjusted covariates by multivariate regression analysis and found that TIVA correlated independently with lower postoperative pulmonary complications. (Odds ratio 0.41, *p* = 0.031)(Table 4) Therefore, patients who received TIVA in perioperative anesthesia management may benefit from less fluid resuscitation and may be less likely to develop pulmonary complications.

Propofol has a central role in total intravenous anesthesia (TIVA). In addition its ability to maintain vascular resistance, propofol has been suggested to modulate the inflammatory response[12] and to attenuate endotoxin-induced endothelia cell injury[13] during acute lung injury. Therefore, for patients whose lung compliance has deteriorated because of general anesthesia and prolonged ventilator support, propofol is considered to have a protective effect against postoperative pulmonary complication via reduction of the systemic inflammatory response. The use of inhalation and intravenous anesthetics in ischemia-reperfusion injury[14], ischemic preconditioning or inflammatory response of the lungs[15] remains controversial.

Some researchers may be concerned about the possibility of metabolic acidosis after prolonged propofol infusion, such as propofol infusion syndrome (PRIS), which would cause damage to a fresh anastomosis flap and increase. Although there is an association between PRIS and propofol infusion at doses higher than 4 mg.kg^-1^.h^-1^ when duration of use is greater than 48 h [16], the target effect-site concentration of the present study was 3.0~5.0mcg‧ml^-1^ and the mean anesthesia duration was only 11.40± 2.40 hours. In this sudy, there was no difference in surgical complication rate between the two groups. TIVA has been applied in many kinds of surgery without the development of PRIS [17, 18]. The main limitations of this study were its retrospective design and small sample size. Because this was a retrospective analysis, no causality could be inferred from our results. Future prospective controlled trials are needed to clarify the beneficial effect and causal associations of different types of anesthesia.

In conclusion, patients who received TIVA had a lower fluid requirement during free flap surgery; hence the pulmonary complications were reduced by nearly 60%. Anesthetics such as propofol may be able to reduce the occurrence of pulmonary complications without significant difference in surgical complication in free flap surgery for head and neck cancer.
